# Supplementary material for: Detecting Sepsis in Patients with Severe Subarachnoid Hemorrhage during Critical Care
Source: J Clin Med. 2022 Jul 21;11(14):4229. doi: 10.3390/jcm11144229 (PMC9319068; doi:10.3390/jcm11144229)
Supplement: Supplementary file 1 [file jcm-11-04229-s001.zip › jcm-1749900-supplementary.pdf]

Supplemental S1

Systemic inflammatory response syndrome (SIRS) score

Awarding one point each for the presence of one of the four designated states:

- fever (at least 38°C) or hypothermia (36°C or less);
- tachycardia: Heart rate at least 90/min;
- tachypnea (least 20/min) or hyperventilation (P_aCO_2 4.3 kPa or less/ 33 mmHg or less);
- leukocytosis (at least 12000/mm^3) or leukopenia (4000/mm^3 or less).

The score is evaluated as positive if a total of at least two points out of a maximum of 4 points are witnessed.
